# 

**Published:** 1889-05-18

**Authors:** 


PRICE ONE PENNY.
No. 138, Vol. VI.—May 18th, 1889.
[Registered at the General Post Office as a Newspaper.]

Per Annum, 6s. 6d., post free.
Monthly Parts, 6d.; post free, 7½d.
Ditto per Annum, 7s. 6d., post free.

